# 3-Hy­droxy-1,2-dimeth­oxyxanthone

**DOI:** 10.1107/S160053681102160X

**Published:** 2011-06-18

**Authors:** Hui-Ping Xiong, Zhi-Jun Wu, Fa-Tang Chen, Dong-Sheng Chen

**Affiliations:** a Department of Mathematics and Physics, Shanghai University of Electric Power, Shanghai 200090, People’s Republic of China; b Department of Pharmacy, Changzheng Hospital, Second Military Medical University, Shanghai 200003, People’s Republic of China

## Abstract

The title compound (systematic name: 3-hy­droxy-1,2-dimeth­oxy-9*H*-xanthen-9-one), C_15_H_12_O_5_, was isolated from *Polygala arillata*. The tricyclic unit is essentially planar (r.m.s. deviation = 0.039 Å). In the crystal, the mol­ecules form stacks along the *a* axis. Inter­molecular O—H⋯O hydrogen bonds link the mol­ecules into chains parallel to [010].

## Related literature

For general background to the title compound and the plant *Polygala arillata*, see: Corrêa *et al.* (1970 ▶); De Oliveira *et al.* (1968 ▶); Dominguez *et al.* (1990 ▶); Gottlieb *et al.* (1970 ▶); Jiangshu New Medicinal College (1977 ▶); Li *et al.* (1999 ▶); Lin *et al.* (2005 ▶); Miao *et al.* (1996 ▶, 1997 ▶).
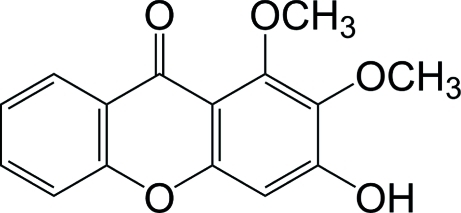


## Experimental

### 

#### Crystal data

C_15_H_12_O_5_
*M* *_r_* = 272.25
Triclinic, 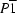

*a* = 7.338 (2) Å
*b* = 7.824 (3) Å
*c* = 11.964 (4) Å
α = 94.634 (4)°
β = 93.561 (4)°
γ = 115.027 (4)°
*V* = 616.8 (3) Å^3^
*Z* = 2
Mo *K*α radiation
μ = 0.11 mm^−1^
*T* = 293 K
0.15 × 0.12 × 0.10 mm

#### Data collection

Bruker SMART APEX CCD area-detector diffractometer
Absorption correction: multi-scan (*SADABS*; Sheldrick, 1996 ▶) *T*_min_ = 0.984, *T*_max_ = 0.989
2562 measured reflections
2126 independent reflections
1697 reflections with *I* > 2σ(*I*)
*R*_int_ = 0.037

#### Refinement

*R*[*F* ^2^ > 2σ(*F* ^2^)] = 0.063
*wR*(*F* ^2^) = 0.190
*S* = 1.07
2126 reflections
184 parameters
H-atom parameters constrained
Δρ_max_ = 0.23 e Å^−3^
Δρ_min_ = −0.32 e Å^−3^

### 

Data collection: *SMART* (Bruker, 1997 ▶); cell refinement: *SAINT* (Bruker, 1997 ▶); data reduction: *SAINT*; program(s) used to solve structure: *SHELXS97* (Sheldrick, 2008 ▶); program(s) used to refine structure: *SHELXL97* (Sheldrick, 2008 ▶); molecular graphics: *SHELXTL* (Sheldrick, 2008 ▶); software used to prepare material for publication: *SHELXTL*.

## Supplementary Material

Crystal structure: contains datablock(s) I, a10401a. DOI: 10.1107/S160053681102160X/yk2009sup1.cif

Structure factors: contains datablock(s) I. DOI: 10.1107/S160053681102160X/yk2009Isup2.hkl

Supplementary material file. DOI: 10.1107/S160053681102160X/yk2009Isup3.cml

Additional supplementary materials:  crystallographic information; 3D view; checkCIF report

## Figures and Tables

**Table 1:** Hydrogen-bond geometry (Å, °)

*D*—H⋯*A*	*D*—H	H⋯*A*	*D*⋯*A*	*D*—H⋯*A*
O5—H5⋯O2^i^	0.82	1.90	2.713 (3)	169

Symmetry code: (i) 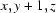.

**Fig. 1.**
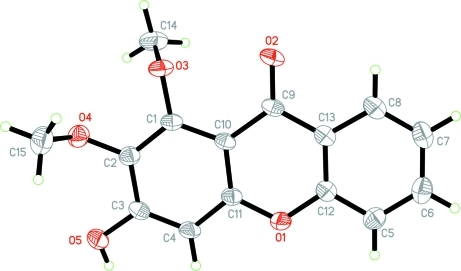
The molecular structure of the title compound, showing the atom-labelling scheme and displacement ellipsoids drawn at the 30% probability level.

## supplementary crystallographic information

### Comment

*Polygala arillata* Buch-Ham is mainly distributed in south-west of China. The roots of *Polygala arillata* has been used in Chinese folk medicine to treat expectorant, hepatitis, pneumonia and rheumatism (Jiangshu New Medicinal College, 1977). Some chemical constituents of this plant have been reported previously (Miao *et al.*, 1996, 1997; Li *et al.*, 1999). Our chemical investigation of this plant for bioactive components resulted in the isolation of the title compound, which was previously obtained from *Kielmeyera rupestris* (Corrêa *et al.*, 1970), *Kielmeyera speciosa* (De Oliveira *et al.*, 1968, Gottlieb *et al.*, 1970), *Polygala nitida* (Dominguez *et al.*, 1990) and *Polygala fallax* (Lin *et al.*, 2005). Herein we report the crystal structure determination of the title compound.

### Experimental

Three 5 kg portions of dry powdered stem bark of *Polygala arillata* were refluxed for 1 h with 95% ethanol (50*L*). After removal of ethanol under reduced pressure, the extract was suspended in water and then partitioned with chloroform, ethyl acetate and n-butanol. The chloroform soluble fraction (50 g) was subjected to silica gel column chromatography using gradient elution (petroleum ether/acetone, 10:1 to 2:1, *v*/*v*). 3-hydroxy-1,2-dimethoxyxanthone was obtained from the fraction eluted by 3:1 petroleum ether/acetone ratio. Single crystals suitable for X-ray diffraction analysis were grown by slow evaporation of acetone solution at room temperature.

### Refinement

The hydroxyl H atoms attached to O5 was located by a difference Fourier map and refined isotropically with a restrained O–H distance 0.82 Å. The remaining H atoms were placed in calculated positions with C—H distances in the range 0.93–0.98 Å. The *U*ĩso~ values were set equal to 1.2*U*eq (C,*O*) for methyl and hydroxyl H atoms and 1.5*U*eq(C) for the remaining H atoms.

### Crystal data

**Table:**

C_15_H_12_O_5_	*Z* = 2
*M**_r_* = 272.25	*F*(000) = 284
Triclinic, *P*1	*D*_x_ = 1.466 Mg m^−^^3^
Hall symbol: -P 1	Mo *K*α radiation, λ = 0.71073 Å
*a* = 7.338 (2) Å	Cell parameters from 804 reflections
*b* = 7.824 (3) Å	θ = 2.9–27.3°
*c* = 11.964 (4) Å	µ = 0.11 mm^−^^1^
α = 94.634 (4)°	*T* = 293 K
β = 93.561 (4)°	Block, colourless
γ = 115.027 (4)°	0.15 × 0.12 × 0.10 mm
*V* = 616.8 (3) Å^3^	

### Data collection

**Table:**

Bruker SMART APEX CCD area-detector diffractometer	2126 independent reflections
Radiation source: fine-focus sealed tube	1697 reflections with *I* > 2σ(*I*)
graphite	*R*_int_ = 0.037
φ and ω scans	θ_max_ = 25.0°, θ_min_ = 1.7°
Absorption correction: multi-scan (*SADABS*; Sheldrick, 1996)	*h* = −6→8
*T*_min_ = 0.984, *T*_max_ = 0.989	*k* = −9→5
2562 measured reflections	*l* = −13→14

### Refinement

**Table:**

Refinement on *F*^2^	Primary atom site location: structure-invariant direct methods
Least-squares matrix: full	Secondary atom site location: difference Fourier map
*R*[*F*^2^ > 2σ(*F*^2^)] = 0.063	Hydrogen site location: inferred from neighbouring sites
*wR*(*F*^2^) = 0.190	H-atom parameters constrained
*S* = 1.07	*w* = 1/[σ^2^(*F*_o_^2^) + (0.1114*P*)^2^ + 0.1836*P*] where *P* = (*F*_o_^2^ + 2*F*_c_^2^)/3
2126 reflections	(Δ/σ)_max_ < 0.001
184 parameters	Δρ_max_ = 0.23 e Å^−^^3^
0 restraints	Δρ_min_ = −0.32 e Å^−^^3^

### Special details

**Table:**

Geometry. All e.s.d.'s (except the e.s.d. in the dihedral angle between two l.s. planes) are estimated using the full covariance matrix. The cell e.s.d.'s are taken into account individually in the estimation of e.s.d.'s in distances, angles and torsion angles; correlations between e.s.d.'s in cell parameters are only used when they are defined by crystal symmetry. An approximate (isotropic) treatment of cell e.s.d.'s is used for estimating e.s.d.'s involving l.s. planes.
Refinement. Refinement of *F*^2^ against ALL reflections. The weighted *R*-factor *wR* and goodness of fit *S* are based on *F*^2^, conventional *R*-factors *R* are based on *F*, with *F* set to zero for negative *F*^2^. The threshold expression of *F*^2^ > 2σ(*F*^2^) is used only for calculating *R*-factors(gt) *etc*. and is not relevant to the choice of reflections for refinement. *R*-factors based on *F*^2^ are statistically about twice as large as those based on *F*, and *R*- factors based on ALL data will be even larger.

### Fractional atomic coordinates and isotropic or equivalent isotropic displacement parameters (Å^2^)

**Table:**

	*x*	*y*	*z*	*U*_iso_*/*U*_eq_	
O1	0.2867 (3)	0.6977 (2)	0.52398 (13)	0.0473 (5)	
O2	0.2172 (3)	0.1946 (2)	0.35370 (16)	0.0603 (6)	
O3	0.2629 (2)	0.3673 (2)	0.15973 (14)	0.0498 (5)	
O4	0.2920 (3)	0.6826 (2)	0.06394 (14)	0.0544 (5)	
O5	0.3176 (3)	0.9927 (2)	0.19396 (15)	0.0592 (6)	
H5	0.2921	1.0654	0.2365	0.089*	
C1	0.2721 (3)	0.5208 (3)	0.22752 (19)	0.0396 (5)	
C2	0.2933 (3)	0.6818 (3)	0.17897 (19)	0.0434 (6)	
C3	0.3077 (3)	0.8442 (3)	0.2474 (2)	0.0446 (6)	
C4	0.3094 (3)	0.8437 (3)	0.3620 (2)	0.0444 (6)	
H4	0.3253	0.9520	0.4077	0.053*	
C5	0.2517 (4)	0.5696 (4)	0.6956 (2)	0.0534 (6)	
H5A	0.2708	0.6866	0.7317	0.064*	
C6	0.2189 (4)	0.4191 (4)	0.7557 (2)	0.0599 (7)	
H6	0.2148	0.4343	0.8333	0.072*	
C7	0.1919 (4)	0.2451 (4)	0.7025 (2)	0.0599 (7)	
H7	0.1689	0.1442	0.7444	0.072*	
C8	0.1988 (4)	0.2210 (4)	0.5886 (2)	0.0522 (6)	
H8	0.1827	0.1041	0.5537	0.063*	
C9	0.2376 (3)	0.3482 (3)	0.4018 (2)	0.0417 (6)	
C10	0.2654 (3)	0.5149 (3)	0.34488 (19)	0.0376 (5)	
C11	0.2873 (3)	0.6818 (3)	0.4091 (2)	0.0397 (5)	
C12	0.2559 (3)	0.5436 (3)	0.5796 (2)	0.0437 (6)	
C13	0.2302 (3)	0.3713 (3)	0.5240 (2)	0.0422 (6)	
C14	0.0691 (5)	0.2530 (4)	0.0989 (3)	0.0700 (8)	
H14A	−0.0279	0.1977	0.1511	0.105*	
H14B	0.0770	0.1539	0.0496	0.105*	
H14C	0.0278	0.3306	0.0551	0.105*	
C15	0.4879 (5)	0.7613 (5)	0.0279 (3)	0.0741 (9)	
H15A	0.5620	0.8891	0.0637	0.111*	
H15B	0.4772	0.7615	−0.0524	0.111*	
H15C	0.5575	0.6865	0.0481	0.111*	

### Atomic displacement parameters (Å^2^)

**Table:**

	*U*^11^	*U*^22^	*U*^33^	*U*^12^	*U*^13^	*U*^23^
O1	0.0617 (10)	0.0392 (9)	0.0448 (9)	0.0267 (8)	0.0019 (8)	0.0002 (7)
O2	0.0869 (14)	0.0343 (9)	0.0675 (12)	0.0328 (9)	0.0145 (10)	0.0052 (8)
O3	0.0571 (10)	0.0413 (9)	0.0539 (10)	0.0275 (8)	−0.0010 (8)	−0.0093 (7)
O4	0.0648 (11)	0.0587 (11)	0.0442 (10)	0.0317 (9)	−0.0002 (8)	0.0053 (8)
O5	0.0896 (14)	0.0429 (10)	0.0591 (11)	0.0397 (10)	0.0147 (10)	0.0137 (8)
C1	0.0373 (11)	0.0346 (11)	0.0480 (13)	0.0186 (9)	0.0005 (9)	−0.0032 (9)
C2	0.0447 (12)	0.0424 (13)	0.0455 (13)	0.0220 (10)	0.0022 (10)	0.0017 (10)
C3	0.0487 (13)	0.0353 (12)	0.0548 (14)	0.0226 (10)	0.0049 (10)	0.0077 (10)
C4	0.0525 (13)	0.0335 (12)	0.0525 (14)	0.0248 (10)	0.0037 (11)	0.0005 (10)
C5	0.0511 (14)	0.0574 (15)	0.0497 (14)	0.0230 (12)	0.0003 (11)	0.0013 (11)
C6	0.0504 (14)	0.0740 (19)	0.0502 (15)	0.0208 (13)	0.0029 (11)	0.0154 (13)
C7	0.0504 (14)	0.0602 (17)	0.0641 (17)	0.0164 (12)	0.0021 (12)	0.0250 (13)
C8	0.0459 (13)	0.0433 (13)	0.0665 (16)	0.0172 (11)	0.0046 (11)	0.0136 (11)
C9	0.0378 (11)	0.0332 (11)	0.0577 (14)	0.0190 (9)	0.0048 (10)	0.0034 (10)
C10	0.0335 (11)	0.0307 (11)	0.0503 (13)	0.0161 (8)	0.0026 (9)	0.0027 (9)
C11	0.0399 (11)	0.0364 (12)	0.0457 (12)	0.0202 (9)	0.0018 (9)	0.0008 (9)
C12	0.0380 (11)	0.0439 (13)	0.0506 (13)	0.0188 (10)	0.0032 (9)	0.0077 (10)
C13	0.0327 (11)	0.0404 (13)	0.0543 (14)	0.0163 (9)	0.0034 (10)	0.0086 (10)
C14	0.0711 (18)	0.0511 (16)	0.078 (2)	0.0234 (13)	−0.0121 (15)	−0.0171 (14)
C15	0.085 (2)	0.083 (2)	0.0597 (17)	0.0379 (17)	0.0243 (16)	0.0156 (15)

### Geometric parameters (Å, °)

**Table:**

O1—C12	1.366 (3)	C5—H5A	0.9300
O1—C11	1.370 (3)	C6—C7	1.383 (4)
O2—C9	1.234 (3)	C6—H6	0.9300
O3—C1	1.368 (3)	C7—C8	1.367 (4)
O3—C14	1.430 (3)	C7—H7	0.9300
O4—C2	1.376 (3)	C8—C13	1.405 (3)
O4—C15	1.415 (3)	C8—H8	0.9300
O5—C3	1.349 (3)	C9—C10	1.463 (3)
O5—H5	0.8200	C9—C13	1.466 (3)
C1—C2	1.383 (3)	C10—C11	1.402 (3)
C1—C10	1.412 (3)	C12—C13	1.386 (3)
C2—C3	1.415 (3)	C14—H14A	0.9600
C3—C4	1.370 (3)	C14—H14B	0.9600
C4—C11	1.379 (3)	C14—H14C	0.9600
C4—H4	0.9300	C15—H15A	0.9600
C5—C6	1.370 (4)	C15—H15B	0.9600
C5—C12	1.391 (4)	C15—H15C	0.9600
			
C12—O1—C11	119.52 (18)	O2—C9—C10	124.6 (2)
C1—O3—C14	114.41 (19)	O2—C9—C13	120.0 (2)
C2—O4—C15	113.4 (2)	C10—C9—C13	115.45 (19)
C3—O5—H5	109.5	C11—C10—C1	116.5 (2)
O3—C1—C2	118.6 (2)	C11—C10—C9	119.1 (2)
O3—C1—C10	120.1 (2)	C1—C10—C9	124.3 (2)
C2—C1—C10	121.2 (2)	O1—C11—C4	114.15 (19)
O4—C2—C1	120.4 (2)	O1—C11—C10	123.0 (2)
O4—C2—C3	119.7 (2)	C4—C11—C10	122.9 (2)
C1—C2—C3	119.8 (2)	O1—C12—C13	122.1 (2)
O5—C3—C4	123.5 (2)	O1—C12—C5	116.1 (2)
O5—C3—C2	116.7 (2)	C13—C12—C5	121.8 (2)
C4—C3—C2	119.7 (2)	C12—C13—C8	117.8 (2)
C3—C4—C11	119.7 (2)	C12—C13—C9	120.7 (2)
C3—C4—H4	120.2	C8—C13—C9	121.4 (2)
C11—C4—H4	120.2	O3—C14—H14A	109.5
C6—C5—C12	118.6 (3)	O3—C14—H14B	109.5
C6—C5—H5A	120.7	H14A—C14—H14B	109.5
C12—C5—H5A	120.7	O3—C14—H14C	109.5
C5—C6—C7	120.9 (3)	H14A—C14—H14C	109.5
C5—C6—H6	119.6	H14B—C14—H14C	109.5
C7—C6—H6	119.6	O4—C15—H15A	109.5
C8—C7—C6	120.3 (2)	O4—C15—H15B	109.5
C8—C7—H7	119.9	H15A—C15—H15B	109.5
C6—C7—H7	119.9	O4—C15—H15C	109.5
C7—C8—C13	120.5 (3)	H15A—C15—H15C	109.5
C7—C8—H8	119.7	H15B—C15—H15C	109.5
C13—C8—H8	119.7		

### Hydrogen-bond geometry (Å, °)

**Table:**

*D*—H···*A*	*D*—H	H···*A*	*D*···*A*	*D*—H···*A*
O5—H5···O2^i^	0.82	1.90	2.713 (3)	169

Symmetry codes: (i) *x*, *y*+1, *z*.
